# Combined Effect of Nitrate Levels and Diabetes on the Risk of Severe Periodontitis: A Cross-sectional Study

**DOI:** 10.3290/j.ohpd.c_2771

**Published:** 2026-08-06

**Authors:** Yu Li, Xiaolong Zhu, Ling Qiu

**Affiliations:** a Yu Li Assistant Professor, School of Medicine, Anqing Medical College, Anqing 246052, Anhui, China; Associate Chief Physician, Anqing Zhuxiaolong Dental Hospital, Anqing 246003, Anhui, China; participated in the study conceptualisation, wrote the original draft, reviewed and edited the methodology, and conducted the formal analysis.; b Xiaolong Zhu Associate Chief Physician, Anqing Zhuxiaolong Dental Hospital, Anqing 246003, Anhui, China; contributed to the data curation, methodology, and formal analysis.; c Ling Qiu Associate Chief Physician, Anqing Zhuxiaolong Dental Hospital, Anqing 246003, Anhui, China; contributed to the data curation, methodology, and formal analysis.

**Keywords:** diabetes, multiplicative interaction, nitrate levels, periodontitis, risk

## Abstract

**Background:**

Nitrate levels and diabetes are important factors influencing the risk of periodontitis. This study intended to assess the combined impact of urinary nitrate levels and diabetes on periodontitis risk in the general population.

**Methods:**

This cross-sectional study employed the data on individuals aged 30 and above from the 2009–2014 National Health and Nutrition Examination Survey (NHANES) database. The urinary nitrate levels were divided into three groups based on the tertiles (low, median, and high). A multivariate logistic regression model was used to investigate the relationship between urinary nitrate levels and the risk of stage III/IV periodontitis, with stratified analysis conducted according to diabetes. The multiplicative interaction between nitrate levels and diabetes on periodontitis risk was analysed using the likelihood ratio test. A restricted cubic splines curve was utilised to assess the non-linear association.

**Results:**

Among the 3,292 included participants, 841 (19.28%) individuals had stage III/IV periodontitis, while 2,451 (80.72%) did not. Compared with median nitrate levels, high nitrate levels (odds ratio [OR] = 1.58, 95% confidence interval [CI]: 1.12 to 2.22]) were associated with higher odds of stage III/IV periodontitis, but no statistically significant association was observed between low nitrate levels and periodontitis risk (*P* > 0.05). Urinary nitrate levels and diabetes showed a multiplicative interaction on the risk of stage III/IV periodontitis (*P* = 0.020). Specifically, high urinary nitrate levels (OR = 1.68, 95% CI: 1.16 to 2.43) were correlated with a higher risk of periodontitis in non-diabetic individuals, whereas in diabetic individuals, low urinary nitrate levels (OR = 1.66, 95% CI: 1.07 to 2.58) were associated with a higher risk of periodontitis. Moreover, urinary nitrate levels exhibited a statistically significant nonlinear relationship with stage III/IV periodontitis risk in the general population.

**Conclusion:**

High nitrate levels and diabetes exhibit a multiplicative interaction on periodontitis risk. Clinicians may need to monitor both blood glucose and urinary nitrate levels to screen individuals at high risk of periodontitis.

Periodontitis is a chronic inflammatory disease that threatens oral health. It compromises the integrity of the teeth’s supporting structures (gingiva, periodontal ligaments, and alveolar bone), potentially leading to tooth loss and systemic inflammation.^21,43
^ Epidemiological data indicate that over one billion people worldwide were affected by severe periodontitis in 2021, with an age-standardised prevalence rate of 12,498.3 per 100,000 people.^45^ Periodontitis not only has adverse effects on teeth but may also increase the risk of cardiovascular disease, diabetes, rheumatoid arthritis, and cancer in patients, thereby impacting overall health.^15,16,40
^ The severity of periodontitis depends on environmental and host risk factors, with smoking, diabetes, and genetic susceptibility being significant risk factors.^21^ Non-surgical treatments, including antimicrobial and antibiotic agents, have been proven to optimise periodontal treatment outcomes.^10,42
^


Some studies have indicated that nitrates play a crucial role in combating pathogens and maintaining oral microecology,^27,39
^ while pathogenic dental plaque biofilm contributes to the development of periodontitis.^21^ The recycling of nitrates and nitrites to nitric oxide (NO) occurs via the intestinal-salivary cycle, which relies on orally commensal nitrate-reducing bacteria residing on the dorsal surface of the tongue.^25,32
^ The nitrate levels in saliva or gingival crevicular fluid have been reported to be positively correlated with periodontal disease,^38^ and it has been proposed to use them as biomarkers for periodontal disease.^33^ However, the evidence for the relationship between nitrate levels and the risk of periodontitis remains limited. Furthermore, diabetes is a significant risk factor for periodontitis, and periodontitis is also a common complication of diabetes.^17,22,28,46
^ Under diabetic conditions, the production of NO may be insufficient as hyperglycaemia affects NO synthesis by inducing oxidative stress and advanced glycation end products.^4^ Animal studies have found that nitrate supplementation can reduce bacteria associated with dental caries and periodontitis, as well as pathogens linked to insulin resistance and impaired glucose tolerance.^2^ Thus, the present authors hypothesise that nitrate levels and diabetes exert a synergistic interactive effect on the risk of onset of periodontitis. The aim of this study was to explore the independent correlation between nitrate levels and periodontitis risk in the general population and to further clarify the combined influence of nitrate levels and diabetes on the occurrence of periodontitis.

## Methods

### Study Design and Participants

**Fig 1 Fig1:**
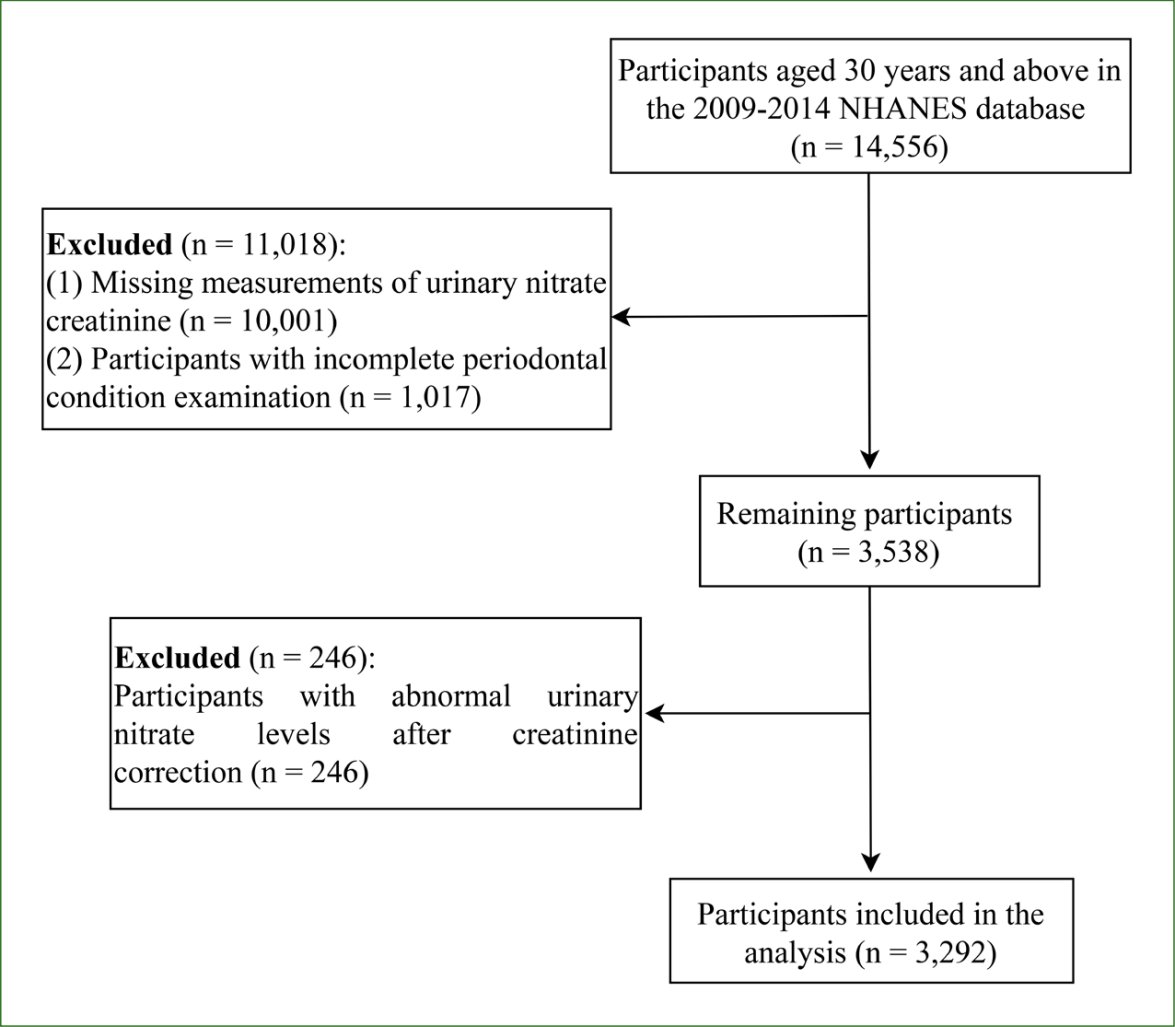
Flowchart of the screening process for the population.

The data for this cross-sectional study were sourced from the 2009–2014 National Health and Nutrition Examination Survey (NHANES) database, representing the most recent data on oral health–periodontal examinations. NHANES is a nationally representative cross-sectional survey conducted every 2 years by the National Center for Health Statistics (NCHS) to assess the health and nutritional status of the general population in the United States (https://www.cdc.gov/nchs/nhanes/). It employs a complex stratified multistage probability design to select survey participants. Data collected by NHANES consist of interviews and physical examinations. The interview component covers demographic, socioeconomic, dietary, and health-related questions, while the examination component includes medical, dental, and physiological measurements. Participants aged 30 and above were initially included. The exclusion criteria were individuals lacking urinary nitrate or creatinine measurement data, individuals with incomplete periodontal status examination data, and individuals with outliers of the creatinine-corrected urinary nitrate levels. The 2009–2014 NHANES database covered 14,556 participants aged 30 years and above. A total of 11,264 participants were excluded, including 10,001 who were lacking urinary nitrate or creatinine measurement data, 1,017 who had incomplete periodontal examination data, and 246 who had abnormal urinary nitrate levels after creatinine correction. Finally, 3,292 eligible participants were included in the analysis (Fig 1). NHANES obtained ethical approval from the Institutional Review Board of the NCHS, with all participants signing written informed consent forms before participation.

### Assessment of Periodontitis

The outcome variable in this study was the occurrence of stage III/IV periodontitis. In the NHANES survey, periodontal examinations were conducted by skilled and calibrated examiners using periodontal probes in a mobile examination centre (MEC) to assess the periodontal conditions of the participants, covering six sites of each tooth (except for the third molars).^5^ Full-mouth periodontal examination is only performed in adults aged 30 years and older, encompassing gingival recession (GR) assessment and periodontal pocket depth measurement. Clinical attachment loss (CAL) is calculated as the sum of the absolute values of probing pocket depth (PPD) and GR. The diagnosis of periodontitis is based on the 2018 criteria established by the European Federation of Periodontology (EFP) and the American Academy of Periodontology (AAP)^31,41
^: (1) periodontitis is diagnosed if CAL ≥ 3 mm is detected in at least two non-adjacent teeth, or buccal or lingual CAL ≥ 3 mm is observed in at least two teeth accompanied by PPD > 3 mm; (2) periodontitis staging: the greatest interdental CAL of 1 to 2 mm loss (stage I), 3 to 4 mm loss (stage II), and ≥ 5 mm loss (stages III to IV). If the greatest PPD is ≥ 6 mm, stage II is reclassified as stage III. If the number of remaining teeth is < 20, stage III is reclassified as stage IV.^6^


### Measurement of Urinary Nitrate

The levels of nitrate in human urine were quantified using ion chromatography coupled with electrospray tandem mass spectrometry. The lower detection limit for urinary nitrate is 700 ng/ml. When the test result was below the limit of detection, the urinary nitrate level of the participant was assigned the value of the detection limit divided by the square root of 2. In this analysis, urinary nitrate levels were corrected for urinary creatinine (corrected nitrate = urinary nitrate [ng/ml] / urinary creatinine [mg/dl]).^24^ The corrected urinary nitrate levels were divided into three groups according to the tertiles: low (< 303.44), median (303.44 to 635.94), and high (≥ 635.94).

### Data Collection

Participants’ data were collected, covering age, sex (male, female), race (White, Black, other), education level (below high school, high school, above high school), poverty income ratio (PIR; < 1.3, ≥ 1.3), smoking status (no, yes), drinking status (no, yes), chronic kidney disease (CKD; no, yes), physical activity (< 750, ≥ 750 metabolic equivalent of task [MET] min/week), the Healthy Eating Index-2020 (HEI-2020), obesity (no, yes), diabetes (no, yes), hypertension (no, yes), cardiovascular disease (CVD; no, yes), dyslipidemia (no, yes), ratio of neutrophils to lymphocytes (NLR), bone loss around teeth (no, yes), frequency of dental floss usage (at least three or fewer than three times/week), dental implant (no, yes), dental caries (no, yes, unknown), urinary nitrate, and urinary creatinine. Smoking status was determined by the question: ‘Have you smoked at least 100 cigarettes in your entire life?’, and drinking status by the question: ‘In any one year, have you had at least 12 drinks of any type of alcoholic beverage? By a drink, I mean a 12-oz beer, a 5-oz glass of wine, or 1.5 oz liquor’. Bone loss around teeth was defined based on the question: ‘Have you ever been told by a dental professional that you had lost bone around your teeth?’ Obesity was defined as a body mass index (BMI) ≥ 30 kg/m^2^. Diabetes was identified based on fasting blood glucose (FBG) ≥ 126 mg/dl or glycated haemoglobin (HbA1c) ≥ 6.5% or self-reported diabetes or the use of hypoglycaemic agents.

### Statistical Analysis

Continuous variables were reported as mean and standard error (SE), while categorical variables were summarised as frequency and percentage (n [%]). Comparison between groups was performed using a weighted *t* test for continuous variables and a Chi-square test for categorical variables. Some covariates had missing values (≤ 5.38%), which were imputed using the multiple imputation method. A difference analysis was conducted before and after imputing missing data (Supplementary Table 1).

**Table 1 Table1:** Characteristics of population

Variable		Total (n = 3,292)	Stage III/IV periodontitis		*P* value
			No (n = 2,451)	Yes (n = 841)	
Age (years), mean (SE)		50.62 (0.36)	49.27 (0.35)	56.31 (0.77)	< 0.001
Sex, n (%)	Male	1,672 (50.08)	1,115 (46.51)	557 (65.02)	< 0.001
	Female	1,620 (49.92)	1,336 (53.49)	284 (34.98)	
Race, n (%)	White	1,419 (67.80)	1,127 (69.84)	292 (59.24)	< 0.001
	Black	715 (11.59)	458 (9.88)	257 (18.77)	
	Other	1,158 (20.61)	866 (20.28)	292 (21.99)	
Education level, n (%)	Below high school	773 (15.19)	473 (12.12)	300 (28.03)	< 0.001
	High school	718 (21.83)	488 (19.73)	230 (30.62)	
	Above high school	1,801 (62.98)	1,490 (68.14)	311 (41.35)	
PIR, n (%)	< 1.3	1,167 (23.90)	763 (20.56)	404 (37.92)	< 0.001
	≥ 1.3	2,125 (76.10)	1,688 (79.44)	437 (62.08)	
Smoking, n (%)	No	1,824 (55.76)	1,473 (59.54)	351 (39.94)	< 0.001
	Yes	1,468 (44.24)	978 (40.46)	490 (60.06)	
Physical activity (MET·min/week), n (%)	< 750	1,479 (40.99)	1,073 (40.50)	406 (43.03)	0.348
	≥ 750	1,813 (59.01)	1,378 (59.50)	435 (56.97)	
HEI-2020, mean (SE)		55.30 (0.30)	55.93 (0.35)	52.67 (0.69)	< 0.001
Drinking, n (%)	No	1,049 (25.29)	781 (25.01)	268 (26.47)	0.511
	Yes	2,243 (74.71)	1,670 (74.99)	573 (73.53)	
CKD, (%)	No	2,778 (87.71)	2,158 (90.16)	620 (77.45)	< 0.001
	Yes	514 (12.29)	293 (9.84)	221 (22.55)	
Obesity, n (%)	No	1,954 (60.66)	1,435 (60.13)	519 (62.84)	0.312
	Yes	1,338 (39.34)	1,016 (39.87)	322 (37.16)	
Diabetes, n (%)	No	2,713 (86.72)	2,084 (88.25)	629 (80.29)	< 0.001
	Yes	579 (13.28)	367 (11.75)	212 (19.71)	
Hypertension, n (%)	No	1,331 (43.30)	1,084 (45.81)	247 (32.79)	< 0.001
	Yes	1,961 (56.70)	1,367 (54.19)	594 (67.21)	
CVD, n (%)	No	3,033 (93.12)	2,304 (94.62)	729 (86.81)	< 0.001
	Yes	259 (6.88)	147 (5.38)	112 (13.19)	
Dyslipidemia, n (%)	No	855 (25.93)	675 (26.62)	180 (23.01)	0.068
	Yes	2,437 (74.07)	1,776 (73.38)	661 (76.99)	
NLR, mean (SE)		2.21 (0.03)	2.20 (0.03)	2.29 (0.06)	0.096
Bone loss around teeth, n (%)	No	2,872 (87.49)	2,192 (90.27)	680 (75.84)	< 0.001
	Yes	420 (12.51)	259 (9.73)	161 (24.16)	
Frequency of dental floss usage, (times/week), n (%)	≤ 3	1,901 (56.43)	1,353 (55.03)	548 (62.27)	0.014
	> 3	1,391 (43.57)	1,098 (44.97)	293 (37.73)	
Dental implant, n (%)	No	3,221 (97.45)	2,391 (97.29)	830 (98.16)	0.330
	Yes	71 (2.55)	60 (2.71)	11 (1.84)	
Dental caries, n (%)	No	133 (2.88)	88 (2.76)	45 (3.40)	0.197
	Yes	1,943 (64.99)	1,474 (65.85)	469 (61.37)	
	Unknown	1,216 (32.13)	889 (31.39)	327 (35.23)	
Urinary nitrate (ng/ml), mean (SE)		49,068.51 (946.95)	48,915.04 (1,168.23)	49,711.21 (1,888.47)	0.741
Urinary creatinine (mg/dl), mean (SE)		112.57 (1.58)	111.65 (2.05)	116.43 (3.44)	0.295
Corrected nitrate, mean (SE)		477.99 (7.34)	479.59 (7.58)	471.29 (14.16)	0.562
Corrected nitrate, n (%)	Low (< 303.44)	884 (23.60)	614 (22.98)	270 (26.19)	0.106
	Median (303.44–635.94)	1,769 (54.32)	1,346 (55.40)	423 (49.80)	
	High (≥ 635.94)	639 (22.08)	491 (21.62)	148 (24.01)	
PIR, poverty income ratio; HEI-2020, Healthy Eating Index-2020; CKD, chronic kidney disease; CVD, cardiovascular disease; NLR, ratio of neutrophils to lymphocytes; corrected nitrate, urinary nitrate/urinary creatinine.					

A weighted univariate logistic regression model was utilised to screen for potential confounders related to stage III/IV periodontitis (Supplementary Table 2), and variables with *P* < 0.05 in the univariate model were adjusted in the multivariate model. A multivariate logistic regression model was applied to evaluate the correlation between urinary nitrate levels and the risk of stage III/IV periodontitis, and the corrections were summarised as odds ratio (OR) and 95% confidence interval (CI). Model 1 was adjusted for age, sex, and race. Model 2 was adjusted for age, sex, race, education level, PIR, smoking status, HEI-2020, CKD, diabetes, hypertension, CVD, bone loss around teeth, and frequency of dental floss usage. The multiplicative interaction between nitrate levels and diabetes on the risk of stage III/IV periodontitis was assessed using the likelihood ratio test.^13^ For this purpose, the multiplicative interaction term between nitrate levels and diabetes was included in Model 2. The *P* value corresponding to the likelihood ratio statistics of the comparison between the ‘multivariate model without multiplicative interaction terms’ and the ‘multivariate model with multiplicative interaction terms’ is less than 0.05, indicating that the multiplicative interaction truly exists. A stratified analysis based on diabetes (no, yes) was performed to evaluate the impact of nitrate levels on the risk of stage III/IV periodontitis. Furthermore, a restricted cubic splines (RCS) curve was employed to assess the non-linear relationship between nitrate levels and the risk of stage III/IV periodontitis in the general population and patients with diabetes,^9^ respectively. Statistical analyses were conducted with SAS 9.4 software (SAS Institute, Cary, NC, USA), while RCS curves were generated using R software (version 4.5.1; Institute for Statistics and Mathematics, Vienna, Austria). *P* < 0.05 was considered statistically significant.

**Table 2 Table2:** The relationship of urinary nitrate levels with the risk of stage III/IV periodontitis

Variable		Model 1		Model 2	
		OR (95% CI)	*P* value	OR (95% CI)	*P* value
Urinary nitrate levels	Low (< 303.44)	1.07 (0.82–1.39)	0.619	0.98 (0.75–1.27)	0.864
	Median (303.44–635.94)	Ref	NA	Ref	NA
	High (≥ 635.94)	1.44 (1.06–1.94)	0.019	1.58 (1.12–2.22)	0.010
NA, not applicable; Ref, reference. Multivariate logistic regression Model 1 was adjusted for age, sex, and race; multivariate logistic regression Model 2 was adjusted for age, sex, race, education level, PIR, smoking status, HEI-2020, CKD, diabetes, hypertension, CVD, bone loss around teeth, and frequency of dental floss usage.					

## Results

### Characteristics of Participants

The characteristics of the included 3,292 participants are listed in Table 1. Among these participants, 841 (19.28%) had stage III/IV periodontitis, while 2,451 (80.72%) did not. The mean age of participants was 50.62 ± 0.36 years, with 1,672 (50.08%) men and 579 (13.28%) individuals with diabetes. The mean urinary nitrate levels after creatinine correction were 477.99 ± 7.34. Statistically significant differences were observed in age, sex, race, education level, PIR, smoking status, HEI-2020, diabetes, hypertension, CVD, bone loss around teeth, and frequency of dental floss usage between individuals with and without stage III/IV periodontitis.

### Association between Urinary Nitrate Levels and the Risk of Stage III/IV Periodontitis

Table 2 lists the relationship of urinary nitrate levels with the risk of stage III/IV periodontitis. Compared with median nitrate levels, high nitrate levels (OR = 1.44 [95% CI: 1.06 to 1.94]) were related to higher odds of stage III/IV periodontitis after adjusting for age, sex, and race, and this association remained statistically significant after adjusting for all confounders (OR = 1.58 [95% CI: 1.12 to 2.22]). However, no statistically significant association was observed between low nitrate levels and the risk of stage III/IV periodontitis (*P* > 0.05).

**Fig 2 Fig2:**
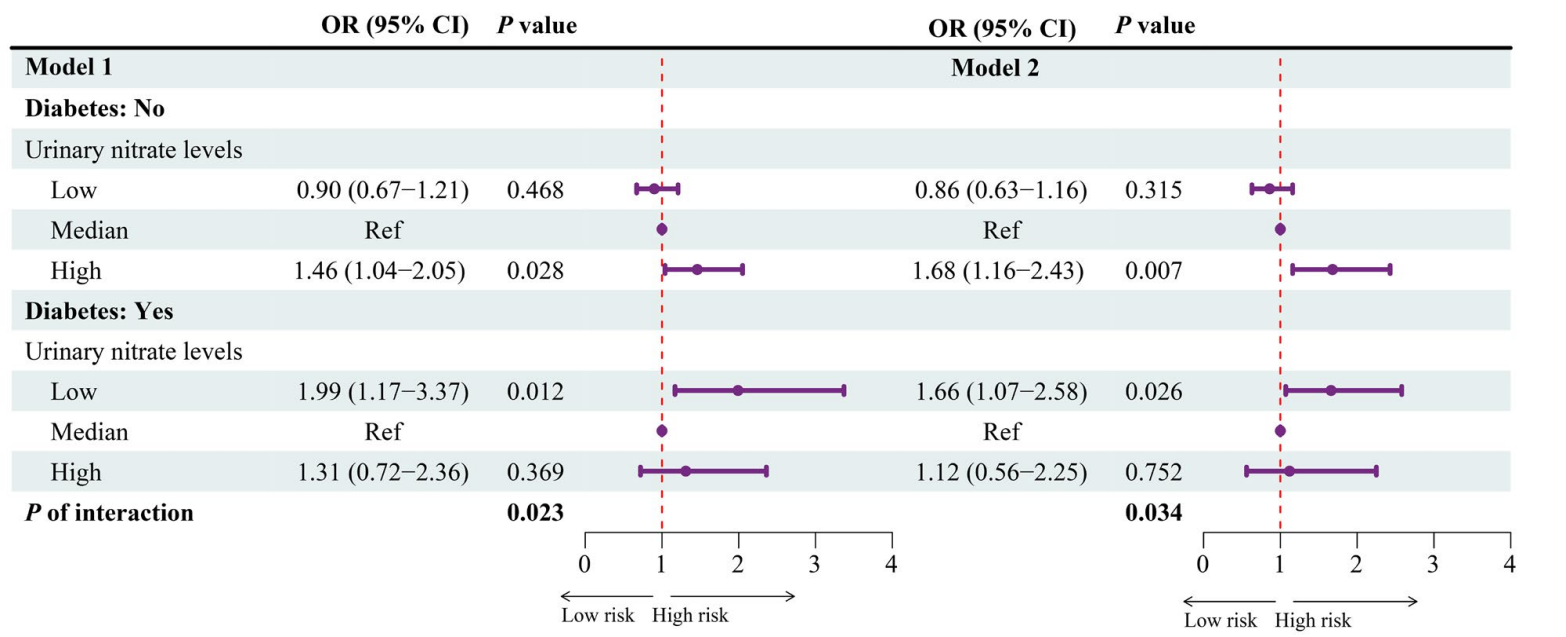
Correlation between urinary nitrate levels and the risk of stage III/IV periodontitis based on diabetes subgroup. Multivariate logistic regression models: Model 1 was adjusted for age, sex, and race; Model 2 was adjusted for age, sex, race, education level, PIR, smoking status, HEI-2020, hypertension, CVD, bone loss around teeth, and frequency of dental floss usage.

The likelihood ratio test demonstrated that the multiplicative interaction between urinary nitrate levels and diabetes was significant for the risk of stage III/IV periodontitis (*P* = 0.020). Figure 2 shows the stratified analysis based on diabetes. High nitrate levels [OR = 1.46 (95% CI: 1.04 to 2.05)] were corrected with higher odds of stage III/IV periodontitis versus median nitrate levels in non-diabetic individuals after adjusting for age, sex, and race, while low nitrate levels [OR = 1.99 (95% CI: 1.17 to 3.37)] were related to higher odds of stage III/IV periodontitis in diabetic individuals. After adjusting for all confounders, high nitrate levels (OR = 1.68 [95% CI: 1.16 to 2.43]) remained related to higher odds of stage III/IV periodontitis in non-diabetic individuals, whereas low nitrate levels (OR = 1.66 [95% CI: 1.07 to 2.58]) remained associated with higher odds of stage III/IV periodontitis in diabetic individuals.

**Fig 3a and b Fig3aandb:**
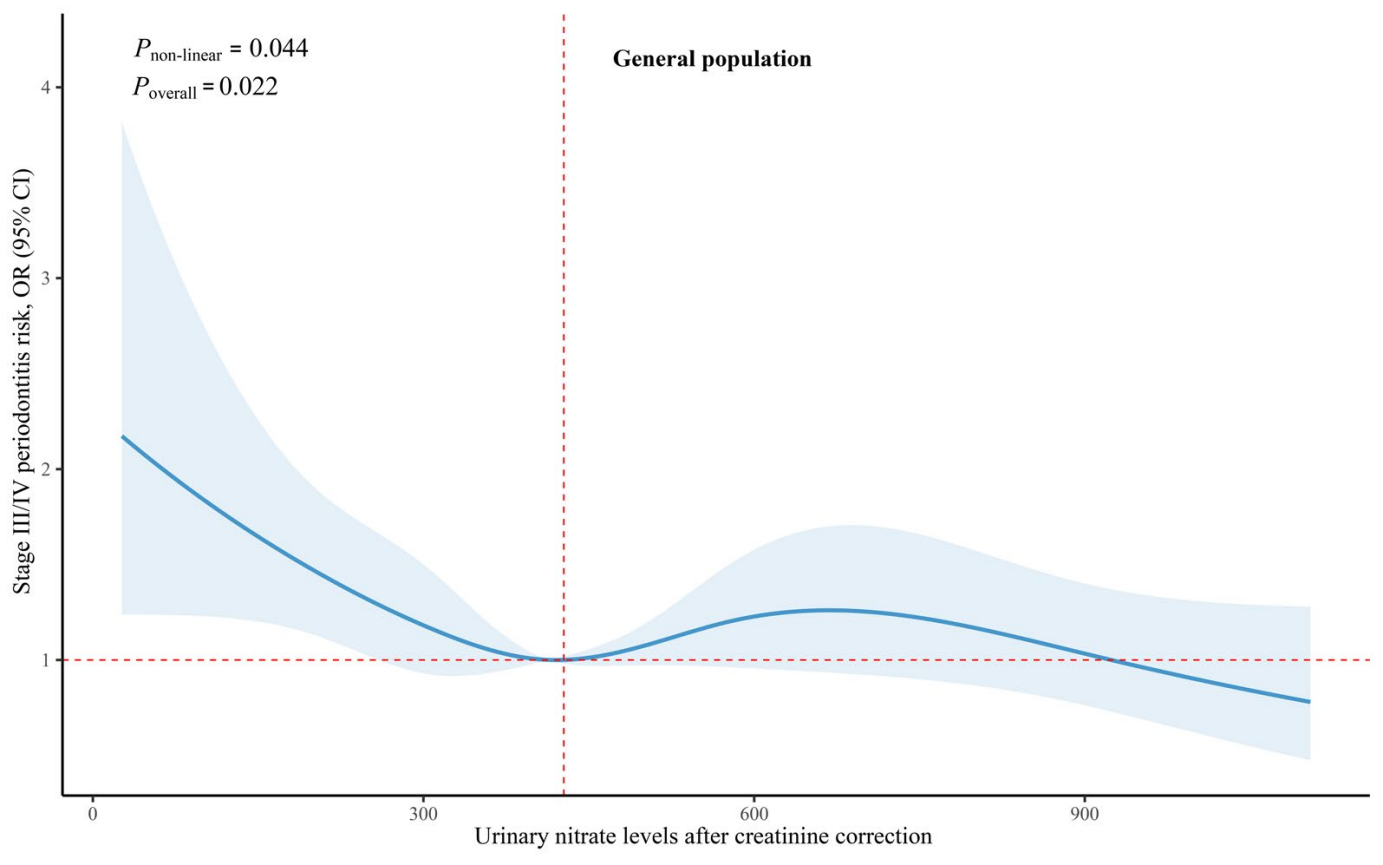

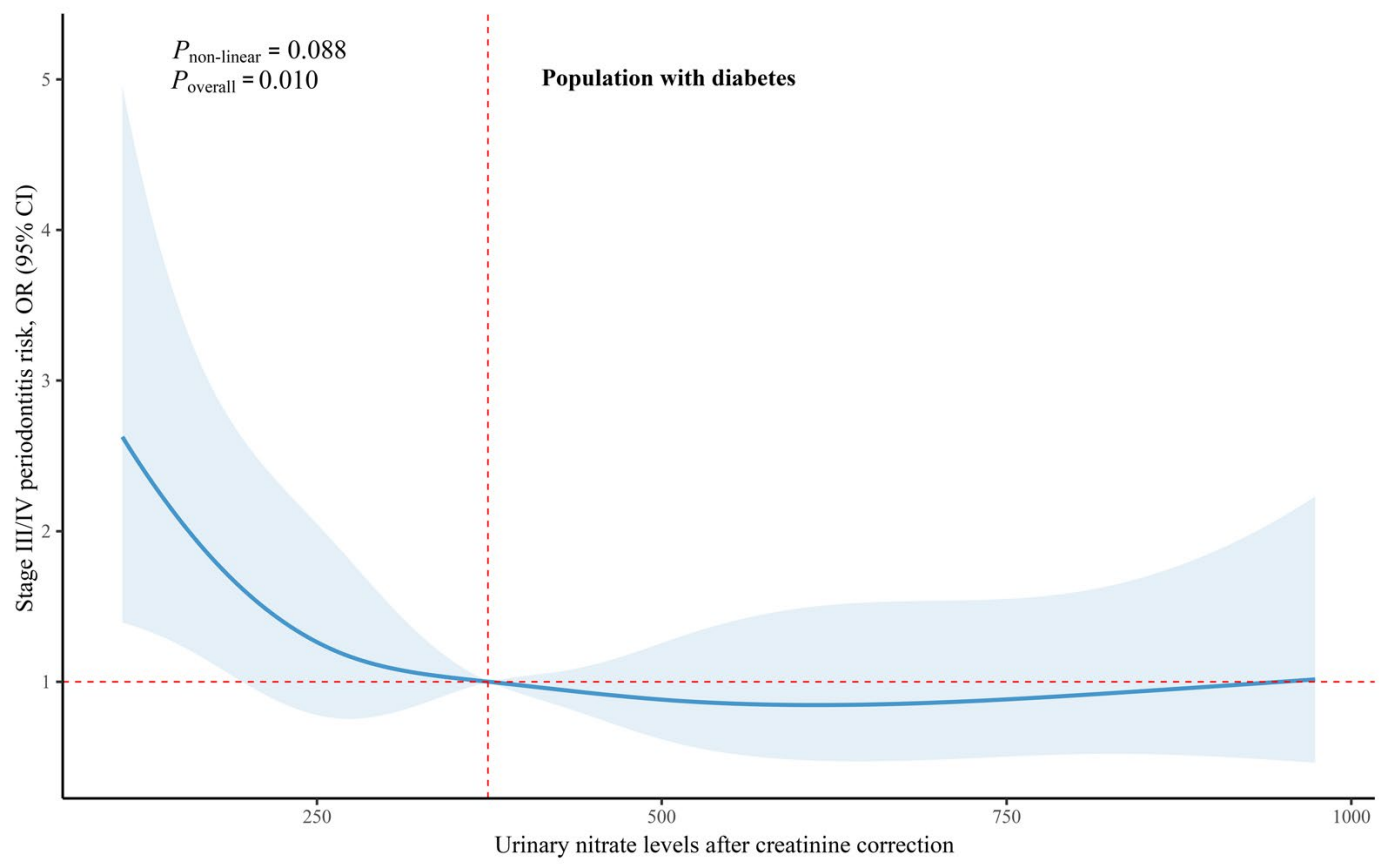
RCS curve of the non-linear relationship between nitrate levels and the risk of stage III/IV periodontitis. General population (a); population with diabetes (b).

Figure 3 presents the RCS curves between nitrate levels and the risk of stage III/IV periodontitis in the general population and diabetic individuals. In the general population, urinary nitrate levels exhibited a statistically significant non-linear relationship with stage III/IV periodontitis risk (*P*
_overall_ = 0.022, *P*
_non-linear_ = 0.044). With the increase of urinary nitrate levels, the risk of stage III/IV periodontitis followed a U-shaped trend: initially decreasing, then increasing, and finally decreasing slightly (Fig 3a). Among diabetic populations, the non-linear relationship between urinary nitrate levels and stage III/IV periodontitis risk was not significant (*P*
_overall_ = 0.010, *P*
_non-linear_ = 0.088). As urinary nitrate levels increased, the risk of stage III/IV periodontitis exhibited an L-shaped trend: initially decreasing, and then gradually stabilising (Fig 3b).

## Discussion

The present study assessed the relationship between urinary nitrate levels and the risk of stage III/IV periodontitis in the general population. High nitrate levels were associated with higher odds of stage III/IV periodontitis, but no statistically significant association was found between low nitrate levels and periodontitis risk. Urinary nitrate levels and diabetes may exhibit a multiplicative interaction on the risk of stage III/IV periodontitis. Specifically, high urinary nitrate levels were correlated with a higher risk of periodontitis in non-diabetic individuals, whereas in diabetic individuals, low urinary nitrate levels were related to a higher risk of periodontitis.

Chronic gingivitis and chronic periodontitis are caused by the oral microbiota (i.e., dental plaque), and immune system dysregulation also plays a major role in the development of periodontitis.^1,7,21
^ The chronic inflammatory process of periodontitis can cause damage to the connective tissue and alveolar bone around the teeth, which is triggered by the microbial components of dental plaque.^18^ Several studies suggested that nitrate levels may be related to the risk of periodontitis.^26,37
^ Diabetes may also play a role in its development.^29^ This study investigated the correlation between urinary nitrate levels and periodontitis risk in the general population, as well as the impact of the interaction between nitrate levels and diabetes on periodontitis risk. The findings revealed that individuals with high nitrate levels had higher odds of stage III/IV periodontitis than individuals with low nitrate levels. Furthermore, the present authors found that in non-diabetic individuals, high nitrate levels were related to a higher risk of periodontitis, whereas in diabetic individuals, low nitrate levels were associated with this. Multivariate interaction analysis also revealed an interaction between nitrate levels and diabetes in influencing the risk of periodontitis. In patients with diabetes, long-term hyperglycaemia can suppress the expression and activity of vascular endothelial NO synthase, leading to impaired endogenous NO synthesis.^23,30
^ Insufficient NO reserves in the body ultimately result in reduced urinary nitrate excretion. Given that individuals with diabetes already exhibit pathological features such as inadequate blood supply to periodontal tissues and reduced regenerative capacity, the NO deficiency reflected by low nitrate levels further exacerbates chronic periodontal inflammation, promotes loss of periodontal attachment and alveolar bone destruction,^30^ and ultimately increases the risk of periodontitis significantly. Among the study population, diabetic participants had a higher proportion of low urinary nitrate levels (34.02% vs. 25.32%) and a lower proportion of high urinary nitrate levels (14.85% vs. 20.38%) relative to non-diabetic individuals (Supplementary Fig 1). In addition, periodontal infection may influence diabetes progression by elevating systemic proinflammatory mediators, which in turn exacerbate insulin resistance. The present study may provide evidence of the correlation between nitrate levels, diabetes, and the risk of periodontitis.

Inorganic nitrates are present in vegetables, particularly green leafy vegetables. Nitrates in food are absorbed through the upper intestine and enter the circulation. Most absorbed nitrates are excreted in urine, but up to 25% are actively extracted by salivary glands and secreted into saliva.^11,34
^ The nitrates in saliva are reduced to nitrites by commensal bacteria and re-enter the circulation via intestinal absorption. Circulating nitrite is reduced to NO by vascular nitrite reductase.^11,14
^ NO plays a significant role in the biological activity and function of the endothelium by regulating vascular tension, inflammation, cell proliferation, and thrombosis.^12^ Animal studies showed that the levels of NO in saliva and plasma were both elevated in the periodontitis group.^8,44
^ Following dietary intake of nitrate-rich foods, the nitrate level in saliva remains elevated for several hours and is influenced by the nitrate dose, time since ingestion, and water intake.^3,36
^ Previous studies have reported that the intake of a diet rich in nitrates (e.g., lettuce juice) can increase the nitrate levels in saliva and reduce chronic gingivitis.^19,20
^ This appears to contradict the present findings that high nitrate levels were associated with higher odds of periodontitis. However, while NO exhibits positive effects as an antimicrobial agent, it is typically associated with the destruction of dental tissues.^33^ Nitrates can stimulate the ecology of the oral microbiota,^35^ reducing gingival inflammation in patients with chronic gingivitis, but they also elevate levels of certain periodontitis-associated bacteria such as *Neisseria subflava* and *R. mucilaginosa*.^37^ This may explain the association between high nitrate levels and higher odds of periodontitis observed in the present results; however, the exact mechanism by which nitrate levels influence the risk of periodontitis may require further research.

This analysis is the first to explore the relationship between nitrate levels and periodontitis risk, and further analysed the impact of the interaction between nitrate and diabetes on periodontitis, providing additional evidence for periodontitis management. The representative large sample data of NHANES ensure the reliability of the analysis results. However, several limitations should be noted. First, the cross-sectional study design of this research means causal inferences cannot be made between nitrate levels and the risk of periodontitis, and the possibility of reverse causality between them cannot be ruled out. Second, the analysed data were extracted from the US population. Due to differences in dietary patterns and lifestyle habits, the generalisability of these findings to other populations requires further validation. Third, although numerous confounders were considered, some potential confounders (e.g., periodontal treatment-subgingival scaling) remain unaccounted for due to database limitations. Fourth, the diagnosis of stage III/IV periodontitis in this study was primarily based on clinical periodontal indices available in the NHANES database, including CAL and PPD, which may have led to misclassification. However, NHANES 2009–2014 employed a periodontal examination protocol that assessed the entire dentition at six sites per tooth, providing relatively comprehensive clinical periodontal measurements for epidemiological classification.

## Conclusions

High nitrate levels were associated with higher odds of stage III/IV periodontitis in the general population. High nitrate levels and diabetes exhibit a multiplicative interaction on periodontitis risk. Clinicians are advised to detect blood glucose and urinary nitrate levels together in oral health screening. Targeted periodontal prevention and interventions for people with hyperglycaemia and high urinary nitrate may reduce the burden of periodontal disorders. Future research may require prospective data to further validate the combined effects of nitrate levels and diabetes on periodontitis risk, as well as to explore the underlying mechanisms involved.

### Acknowledgements

This work was supported by the Domestic Visiting Scholar Programme for Young Core Faculty Members of Anhui Province (grant number JNFX2024109), the Quality Engineering Project of Anhui Province (grant number 2024cyzyq061), and the Natural Science Research Project of the Department of Education of Anhui Province (grant number 2025AHGXZK30799).

## References

### Appendix

**Table A1 tableA1:** Difference analysis before and after imputation of missing data

Variables		Percentage of missing values, n (%)	After imputation	Before imputation	Statistics	*P* value
Education level, n (%)		3 (0.09%)	NA	NA	χ^2^ = 2.906	0.234
	Below high school	NA	773 (15.19)	773 (15.20)	NA	NA
	High school	NA	718 (21.83)	718 (21.85)	NA	NA
	Above high school	NA	1,801 (62.98)	1,798 (62.95)	NA	NA
Physical activity (MET·min/week), n (%)		1 (0.03%)	NA	NA	χ^2^ = 0.991	0.320
	< 750	NA	1,479 (40.99)	1,478 (40.98)	NA	NA
	≥ 750	NA	1,813 (59.01)	1,813 (59.02)	NA	NA
HEI-2020, mean (SE)		177 (5.38%)	55.30 (0.30)	55.29 (0.31)	t = 0.23	0.816
Bone loss around teeth, n (%)		48 (1.46%)	NA	NA	χ^2^ = 0.054	0.816
	No	NA	2,872 (87.49)	2,830 (87.48)	NA	NA
	Yes	NA	420 (12.51)	414 (12.52)	NA	NA
Frequency of dental floss usage, n (%)		27 (0.82%)	NA	NA	χ^2^ = 1.235	0.266
	≤ 3 times/week	NA	1,901 (56.43)	1,887 (56.51)	NA	NA
	> 3 times/week	NA	1,391 (43.57)	1,378 (43.49)	NA	NA
NLR, mean (SE)		113 (3.43%)	2.21 (0.03)	2.21 (0.03)	t = 0.91	0.368
HEI-2020, Healthy Eating Index-2020; NA, not applicable; NLR, ratio of neutrophils to lymphocytes; SE, standard error.						

**Table A2 TableA2:** Screening for potential confounders related to stage III/IV periodontitis

Variables		Univariate logistic regression	
		OR (95% CI)	*P* value
Age		1.04 (1.03–1.05)	< 0.001
Sex	Male	Ref	NA
	Female	0.47 (0.37–0.59)	< 0.001
Race	White	Ref	NA
	Black	2.24 (1.70–2.95)	< 0.001
	Other	1.28 (0.91–1.79)	0.150
Education level	Below high school	Ref	NA
	High school	0.67 (0.51–0.88)	0.005
	Above high school	0.26 (0.20–0.34)	< 0.001
PIR	< 1.3	Ref	NA
	≥ 1.3	0.42 (0.33–0.54)	< 0.001
Smoking	No	Ref	NA
	Yes	2.21 (1.76–2.78)	< 0.001
Physical activity	< 750	Ref	NA
	≥ 750	0.90 (0.72–1.13)	0.352
HEI-2020		0.98 (0.97–0.99)	< 0.001
Drinking	No	Ref	NA
	Yes	0.93 (0.73–1.17)	0.515
CKD	No	Ref	NA
	Yes	2.67 (1.99–3.57)	< 0.001
Obesity	No	Ref	NA
	Yes	0.89 (0.71–1.12)	0.318
Diabetes	No	Ref	NA
	Yes	1.84 (1.30–2.63)	0.001
Hypertension	No	Ref	NA
	Yes	1.73 (1.36–2.20)	< 0.001
CVD	No	Ref	NA
	Yes	2.68 (1.81–3.96)	< 0.001
Dyslipidemia	No	Ref	NA
	Yes	1.21 (0.98–1.50)	0.073
NLR		1.07 (0.99–1.15)	0.070
Bone loss around teeth	No	Ref	NA
	Yes	2.96 (2.32–3.76)	< 0.001
Frequency of dental floss usage	≤ 3 times/week	Ref	NA
	> 3 times/week	0.74 (0.58–0.95)	0.018
Dental implant	No	Ref	NA
	Yes	0.67 (0.29–1.54)	0.342
Dental caries	No	Ref	NA
	Yes	0.76 (0.50–1.15)	0.187
	Unknown	0.91 (0.58–1.44)	0.683
CI, confidence interval; CKD, chronic kidney disease; CVD, cardiovascular disease; HEI-2020, the Healthy Eating Index-2020; NA, not applicable; NLR, the ratio of neutrophils to lymphocytes; OR, odds ratio; PIR, poverty income ratio; Ref, reference.			

## References

[ref1] Ahmad P, Slots J, Siqueira WL (2025). Serum cytokines in periodontal diseases. Periodontol 2000.

[ref2] Bahadoran Z, Mirmiran P, Carlström M, Ghasemi A (2021). Inorganic nitrate: A potential prebiotic for oral microbiota dysbiosis associated with type 2 diabetes. Nitric Oxide.

[ref3] Burleigh M, Liddle L, Muggeridge DJ (2019). Dietary nitrate supplementation alters the oral microbiome but does not improve the vascular responses to an acute nitrate dose. Nitric Oxide.

[ref4] Capellini VK, Celotto AC, Baldo CF (2010). Diabetes and vascular disease: Basic concepts of nitric oxide physiology, endothelial dysfunction, oxidative stress and therapeutic possibilities. Curr Vasc Pharmacol.

[ref6] Chen Y, Lu P, Lin C (2024). Hyperuricemia and elevated uric acid/creatinine ratio are associated with stages III/IV periodontitis: A population-based cross-sectional study (NHANES 2009-2014). BMC Oral Health.

[ref8] Cintra LT, Samuel RO, Azuma MM (2016). Multiple apical periodontitis influences serum levels of cytokines and nitric oxide. J Endod.

[ref9] Desquilbet L, Mariotti F (2010). Dose-response analyses using restricted cubic spline functions in public health research. Stat Med.

[ref11] Fernandes D, Khambata RS, Massimo G (2023). Local delivery of nitric oxide prevents endothelial dysfunction in periodontitis. Pharmacol Res.

[ref14] Ghosh SM, Kapil V, Fuentes-Calvo I (2013). Enhanced vasodilator activity of nitrite in hypertension: Critical role for erythrocytic xanthine oxidoreductase and translational potential. Hypertension.

[ref15] Hajishengallis G (2015). Periodontitis: From microbial immune subversion to systemic inflammation. Nat Rev Immunol.

[ref16] Hajishengallis G, Chavakis T (2021). Local and systemic mechanisms linking periodontal disease and inflammatory comorbidities. Nat Rev Immunol.

[ref17] Holtfreter B, Pitchika V, Aarabi G (2026). Cross-sectional associations of type 2 diabetes with oral conditions: results of the 6th German Oral Health Study (DMS • 6). Quintessence Int.

[ref18] Hussain QA, McKay IJ, Gonzales-Marin C, Allaker RP (2015). Regulation of adrenomedullin and nitric oxide production by periodontal bacteria. J Periodontal Res.

[ref19] Jockel-Schneider Y, Goßner SK, Petersen N (2016). Stimulation of the nitrate-nitrite-NO-metabolism by repeated lettuce juice consumption decreases gingival inflammation in periodontal recall patients: A randomized, double-blinded, placebo-controlled clinical trial. J Clin Periodontol.

[ref20] Jockel-Schneider Y, Schlagenhauf U, Stölzel P (2021). Nitrate-rich diet alters the composition of the oral microbiota in periodontal recall patients. J Periodontol.

[ref21] Kinane DF, Stathopoulou PG, Papapanou PN (2017). Periodontal diseases. Nat Rev Dis Primers.

[ref22] Kudiyirickal MG, Pappachan JM (2024). Periodontitis: An often-neglected complication of diabetes. World J Diabetes.

[ref23] Lalla E, Papapanou PN (2011). Diabetes mellitus and periodontitis: A tale of two common interrelated diseases. Nat Rev Endocrinol.

[ref24] Liu G, Zong G, Dhana K (2017). Exposure to perchlorate, nitrate and thiocyanate, and prevalence of diabetes mellitus. Int J Epidemiol.

[ref25] Lundberg JO, Weitzberg E, Gladwin MT (2008). The nitrate-nitrite-nitric oxide pathway in physiology and therapeutics. Nat Rev Drug Discov.

[ref26] Mazurel D, Carda-Diéguez M, Langenburg T (2023). Nitrate and a nitrate-reducing Rothia aeria strain as potential prebiotic or synbiotic treatments for periodontitis. NPJ Biofilms Microbiomes.

[ref27] Moran SP, Rosier BT, Henriquez FL, Burleigh MC (2024). The effects of nitrate on the oral microbiome: A systematic review investigating prebiotic potential. J Oral Microbiol.

[ref28] Nascimento GG, Leite FRM, Vestergaard P, Scheutz F, López R (2018). Does diabetes increase the risk of periodontitis? A systematic review and meta-regression analysis of longitudinal prospective studies. Acta Diabetol.

[ref29] Negrini TC, Carlos IZ, Duque C, Caiaffa KS, Arthur RA (2021). Interplay among the oral microbiome, oral cavity conditions, the host immune response, diabetes mellitus, and its associated-risk factors-An overview. Front Oral Health.

[ref30] Nibali L, Gkranias N, Mainas G, Di Pino A (2022). Periodontitis and implant complications in diabetes. Periodontol 2000.

[ref32] Pignatelli P, Fabietti G, Ricci A, Piattelli A, Curia MC (2020). How periodontal disease and presence of nitric oxide reducing oral bacteria can affect blood pressure. Int J Mol Sci.

[ref33] Poorsattar Bejeh-Mir A, Parsian H, Akbari Khoram M, Ghasemi N, Bijani A, Khosravi-Samani M (2014). Diagnostic role of salivary and GCF nitrite, nitrate and nitric oxide to distinguish healthy periodontium from gingivitis and periodontitis. Int J Mol Cell Med.

[ref34] Qin L, Liu X, Sun Q (2012). Sialin (SLC17A5) functions as a nitrate transporter in the plasma membrane. Proc Natl Acad Sci U S A.

[ref36] Rosier BT, Palazón C, García-Esteban S, Artacho A, Galiana A, Mira A (2021). A single dose of nitrate increases resilience against acidification derived from sugar fermentation by the oral microbiome. Front Cell Infect Microbiol.

[ref37] Rosier BT, Takahashi N, Zaura E (2022). The importance of nitrate reduction for oral health. J Dent Res.

[ref38] Sánchez GA, Miozza VA, Delgado A, Busch L (2014). Total salivary nitrates and nitrites in oral health and periodontal disease. Nitric Oxide.

[ref39] Schlagenhauf U (2022). On the role of dietary nitrate in the maintenance of systemic and oral health. Dent J (Basel).

[ref40] Sun L, Zheng Q, Hou Y, Lou H (2026). Causal association between systemic diseases and periodontitis: A twoway two-sample Mendelian randomization study. Quintessence Int.

[ref43] Villoria GEM, Fischer RG, Tinoco EMB, Meyle J, Loos BG (2024). Periodontal disease: A systemic condition. Periodontol 2000.

[ref44] Wang Y, Huang X, He F (2019). Mechanism and role of nitric oxide signaling in periodontitis. Exp Ther Med.

[ref45] Wu L, Huang CM, Wang Q, Wei J, Xie L, Hu CY (2025). Burden of severe periodontitis: new insights based on a systematic analysis from the Global Burden of Disease Study 2021. BMC Oral Health.

[ref46] Zheng M, Wang C, Ali A, Shih YA, Xie Q, Guo C (2021). Prevalence of periodontitis in people clinically diagnosed with diabetes mellitus: a meta-analysis of epidemiologic studies. Acta Diabetol.

